# Saccadic Momentum and Facilitation of Return Saccades Contribute to an Optimal Foraging Strategy

**DOI:** 10.1371/journal.pcbi.1002871

**Published:** 2013-01-17

**Authors:** Niklas Wilming, Simon Harst, Nico Schmidt, Peter König

**Affiliations:** 1 Institute of Cognitive Science, University of Osnabrück, Osnabrück, Germany; 2 Department of Informatics, University of Zürich, Zürich, Switzerland; 3 Department of Neurophysiology and Pathophysiology, University Medical Center Hamburg-Eppendorf, Hamburg, Germany; Indiana University, United States of America

## Abstract

The interest in saccadic IOR is funneled by the hypothesis that it serves a clear functional purpose in the selection of fixation points: the facilitation of foraging. In this study, we arrive at a different interpretation of saccadic IOR. First, we find that return saccades are performed much more often than expected from the statistical properties of saccades and saccade pairs. Second, we find that fixation durations before a saccade are modulated by the relative angle of the saccade, but return saccades show no sign of an additional temporal inhibition. Thus, we do not find temporal saccadic inhibition of return. Interestingly, we find that return locations are more salient, according to empirically measured saliency (locations that are fixated by many observers) as well as stimulus dependent saliency (defined by image features), than regular fixation locations. These results and the finding that return saccades increase the match of individual trajectories with a grand total priority map evidences the return saccades being part of a fixation selection strategy that trades off exploration and exploitation.

## Author Summary

Sometimes humans look at the same location twice. To appreciate the importance of this inconspicuous statement you have to consider that we move our eyes several billion (10^9^) times during our lives and that looking at something is a necessary condition to enable conscious visual awareness. Thus, understanding why and how we move our eyes provides a window into our mental life. Here we investigate one heavily discussed aspect of human's fixation selection strategy: whether it inhibits returning to previously fixated locations. We analyze a large data set (more than 550,000 fixations from 235 subjects) and find that, returning to previously fixated locations happens much more often than expected from the statistical properties of eye-movement trajectories. Furthermore, those locations that we return to are not ordinary – they are more salient than locations that we do not return to. Thus, the inconspicuous statement that we look at the same locations twice reveals an important aspect of our strategy to select fixation points: That we trade off exploring our environment against making sure that we have fully comprehended the relevant parts of our environment.

## Introduction

The effect of inhibition of return (IOR) was first described by Posner & Cohen [1]. When (covert) attention is attracted by a peripheral cue, reaction times to a subsequent probe stimulus in the same location depend in an intriguing way on the temporal offset between cue and probe: When the probe follows the cue at temporal offsets shorter than ∼225 ms, fast responses are observed. In contrast, longer offsets (∼225–1500 ms) lead to prolonged response times. In the original experiment, a central cross-had to be fixated continuously, so the inhibitory influence at long stimulus intervals pertained to covert attention. Along similar lines, overt attention—i.e., eye movements—shows the effect of temporal IOR as well. Specifically, the fixation duration before a return saccade is on average longer compared to a saccade that continues in the same direction as the previous one. Unfortunately, several conflicting results make a comprehensive explanation of saccadic IOR and its function difficult. This study aims at a step towards an understanding of these conflicting results by further characterizing the properties of return saccades and by providing a novel view of IOR during viewing of pictures of natural and urban scenes.

But first, we shortly recap some of the discussion surrounding a functional interpretation of IOR. Posner and Cohen hypothesized that IOR might prevent the return of attention to already processed locations. A further investigation by Klein and MacInnes [2] revealed that eye movements are spatially biased away from the last (1-back) and second to last (2-back) fixation locations. This established the interpretation of saccadic IOR not only in the form of a delay, but also in spatial terms as a “foraging facilitator”. That is, the function of saccadic IOR is to direct attention to unexplored parts of the stimulus, thereby fostering optimal foraging behavior. This conjecture subsequently found its way into computational models of fixation selection where saccadic IOR prevents fixating on a location twice [3]–[7].

Whether saccadic IOR supports such a functional “facilitator” role has been heavily discussed. There is conflicting evidence on the spatial properties of return saccades. Several studies [2], [8]–[11] have investigated how often return saccades occur and found, depending on the precise comparison, an elevated or attenuated number of return saccades. Thus, although of crucial importance for the functional interpretation of saccadic IOR, its spatial properties are still hotly debated. There is also mixed evidence on the temporal properties of IOR. Several studies report a significantly prolonged duration of fixation before saccades to the last fixation location [2], [8], [12]. However, [9], [13] reported a general dependency of fixation durations on the angular difference between the previous and the next saccade (termed “saccadic momentum” by Smith and Henderson). They argue that this accounts for parts of temporal IOR but that an additional localized inhibition zone remains. For saccades to the penultimate (2-back) fixation location conflicting evidence is reported whether 2-back return saccades are delayed [2], [8], [9], [11]. In summary, the conflicting evidence of temporal and spatial properties makes it difficult to interpret saccadic IOR as a “foraging facilitator”.

The dominating suggestion in the literature is that IOR supports optimal foraging strategies. This is fueled by the intuition that returning to previously fixated locations is not optimal for foraging because a return saccade does not explore new parts of the environment. Hence, alternating observations of the presence/absence of inhibition of return have been taken as evidence in favor/against an optimal search strategy. However, these arguments are typically based on implicit assumptions regarding an optimal strategy and laboratory experiments with a task where it is difficult to identify the optimal foraging strategy, and therefore not based on direct investigations of fixation selection strategies. Therefore, it is presently unclear whether return fixations, contrary to the assumption that they are non-optimal, can actually be part of an optimal fixation selection strategy under natural conditions. With this in mind, we arrive at the key question of whether return locations are different from other fixation locations. For example, especially salient locations might be more likely to be fixated again, or targets of return saccades might require significantly more time to be comprehended compared to normal fixations. Such findings would suggest that return saccades might actually be due to a fixation selection strategy that needs to find a trade-off between factors such as exploration and comprehension.

We present a thorough investigation of temporal and spatial properties of return saccades by evaluating a large eye-tracking data set compiled from a host of different studies [14]–[17]. We analyze more than half a million fixations collected with natural scenes, urban scenes, fractals and pink noise images from 235 subjects in 5 different studies. These studies employed either free viewing conditions or a delayed patch recognition task. First, we analyze the frequency of 1- and 2-back return saccades and compare them to estimates of the number of return saccades expected from the statistical properties of single saccades and saccade-pairs. We also investigate the temporal properties of return saccades—i.e., if they are preceded by prolonged fixation durations—while paying attention to the effect of saccadic momentum. We then investigate the relationship of return locations to bottom-up saliency (as defined by local image properties). Finally, we investigate the functional role of return saccades to get a better understanding of the functional purpose of saccadic IOR and what exploration strategies could lead to the observed pattern of return saccades.

We arrive at the view that saccadic momentum can fully account for temporal IOR; that return locations are highly salient and warrant increased scrutiny by the human observer; that this scrutiny is implemented by return saccades that are observed more often than expected by chance and by increased fixation durations at return locations; and that these properties of return saccades contribute to an optimal explorative strategy.

## Results

### Spatial Properties of Return Saccades

**Figure 1:**
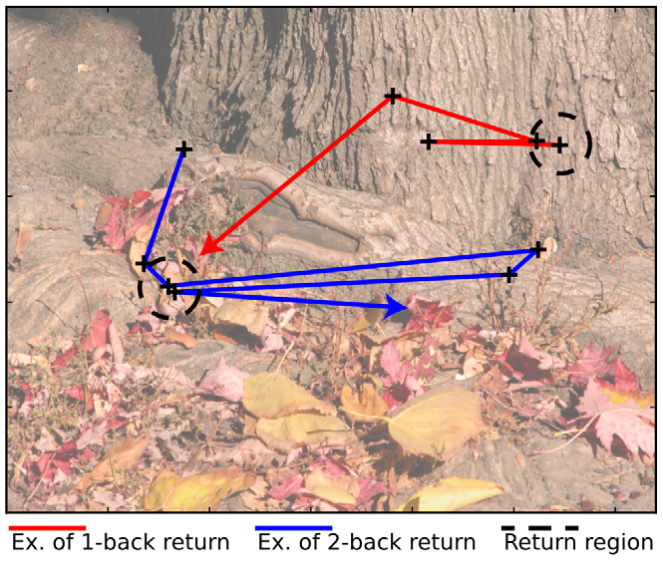
Example image from the category ‘natural scenes’. The red line represents part of a trajectory that contains a 1-back return saccade. The blue trajectory contains a 2-back return saccade. The return region, used as a definition for return saccades for the temporal, saliency, and fixation sampling analysis is marked by the dashed circle.

**Figure 2:**
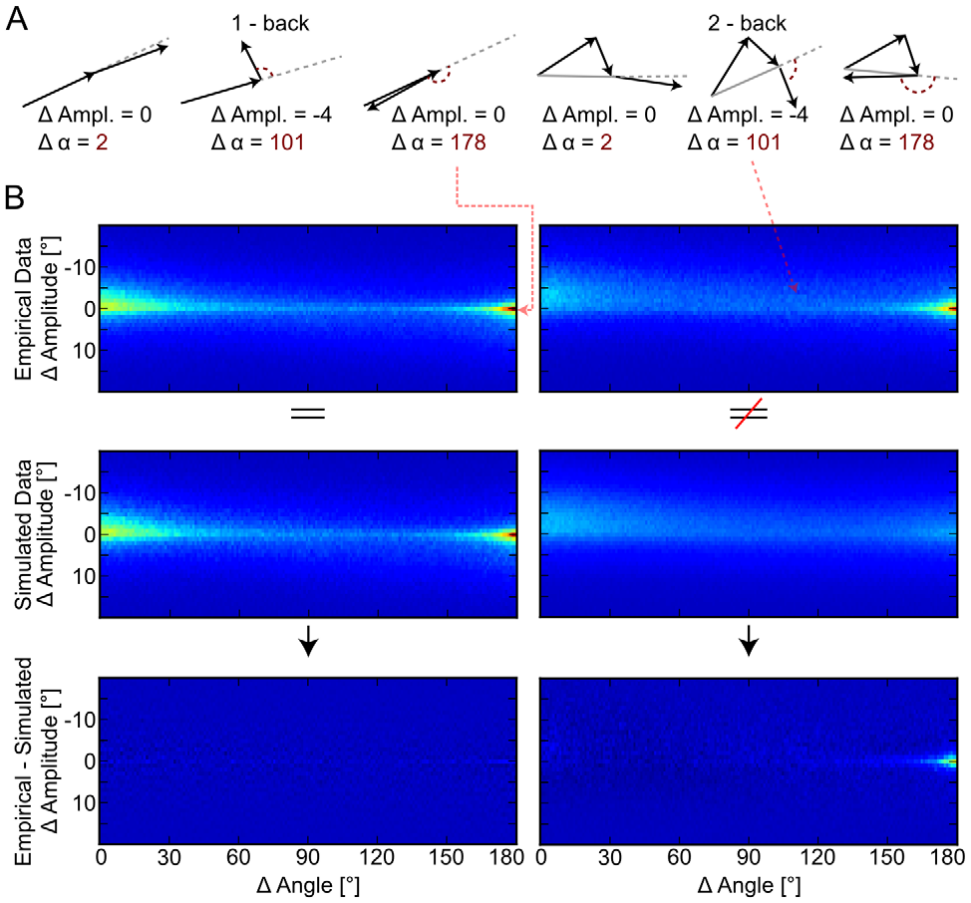
A shows an iconic depiction of forward ( 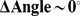
 **), perpendicular (** 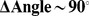 **), and return saccades (** 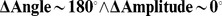 **) in the 1 and 2-back case and their associated angle and amplitude differences.** The first row in B shows the distribution of amplitude and angle differences for empirical 1-back (left) and 2-back (right) saccades. In both cases, a pronounced return saccade peak is observable. The second row shows the same, but for saccades generated with our saccade simulator. Notably, the return peak for 1-back saccades matches the peak in the empirical data while the 2-back return peak is not reproduced. The difference between empirical data and simulator output is shown in the third row (same color scheme as above). The comparison of 2-back saccades shows systematic deviations for return saccades.

**Figure 3:**
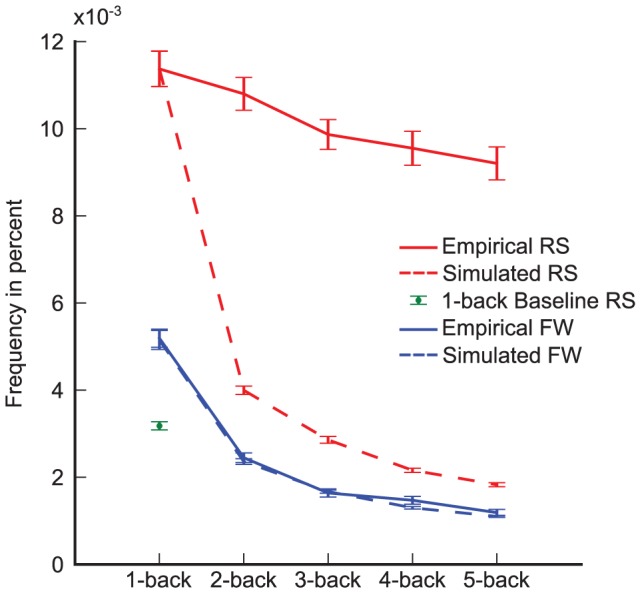
The frequency of return and forward saccades in empirical data and in simulation. For the case of 1-back saccades, the number of empirically observed return saccades (‘Empirical RS’) is larger than expected by chance (‘1-back Baseline RS’) and larger compared to the number of forward saccades (‘Empirical FW’). The simulator reproduces the number of forward (‘Simulated FW’) and return saccades (‘Simulated RS’). In the case of 2-back saccades, we find more return saccades than expected from the statistics of 1-back saccades, while the number of forward saccades is identical to the number of simulated forward saccades. When analyzing the presence of 3- to 5-back saccades, a similar pattern holds. Errorbars are bootstrapped 95% confidence intervals.

We started by investigating how often return saccades occur during viewing of natural scenes. Figure 1 shows an example image with a 1-back (red) and a 2-back (blue) trajectory. Figure 2B (top left row) shows the frequency of saccade pairs with a specific amplitude and angle difference. In this plot, return saccades have a value of 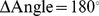 and 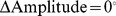. We compared the number of 1-back return saccades to either the number of forward saccades or to a shuffled baseline [8] that preserved the distribution of saccade amplitudes and angles but removed order effects. The shuffled baseline accounts for return saccades due to preferences of saccade angle and amplitude combinations by the oculomotor system, but does not contain return saccades caused by facilitation or inhibition of return. In both cases, we found significantly more return saccades (bootstrapped, 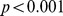, Figure 3, 95% CIs created by bootstrapping per-subject percentages) in the empirical data than in the 1-back baseline. Qualitative inspection of the distribution of angle- and amplitude-differences (see Figure 3B top left row) revealed a sharp peak for return saccades 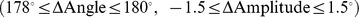 while forward saccades 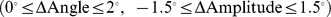 appeared frequently but covered a larger range of amplitude and angle differences. We also observed an asymmetry with respect to amplitude-differences. Forward saccades were often shorter than their preceding saccades (see Figure 2B top left panel). In summary, 1-back return saccades appeared much more often than expected by the distribution of saccade amplitudes and angles, and even more often than forward saccades.

Next we investigated how often 2-back return saccades occur during viewing of natural scenes. While shuffling the order of saccades removes order effects for 1-back return saccades, it does not produce an adequate control distribution for 2-back return saccades. In order for this to be the case, one has to keep all 1-back return saccades due to preferences of the oculomotor system for combinations of angle and amplitudes of two consecutive saccades, but ignore all effects due to preferences of the oculomotor system for angle and amplitudes between three or more consecutive saccades (see Figure 2A, 2-back). We created control trajectories by sampling of saccades from the conditional distribution 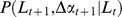 (see Materials and Methods) for each subject. This distribution expresses the probability of a saccade with amplitude 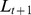 and angle difference 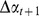 given that the last saccade had amplitude 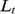. It fully characterizes the angle and amplitude dependencies between two consecutive saccades but does not contain information about 2-back return saccades. To create a trajectory, we randomly drew a saccade from the distribution of first saccades for a given subject and then determined the next saccade's angle and amplitude by sampling from 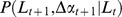. We then iteratively added saccades to the trajectory by sampling new amplitudes and angle differences from 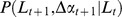, always reusing the last angle and amplitude. We matched the length of the simulated trajectories to the empirically observed lengths'.

The control trajectories reliably reproduced 1-back dependencies and the number of 1-back return saccades in particular, as well as the overall shape of the distribution of angle- and amplitude-differences between consecutive saccades (see Figure 2B, left panels). However, the control trajectories contained fewer 2-back return saccades than observed in the real data (0.0040, bootstrapped CI [0.0038, 0.0042] vs.0.0108, bootstrapped CI [0.0100, 0.0116], Figure 3). The number of 2-back return saccades was much larger than the number of forward saccades (0.0023, CI [0.00211, 0.00244], Figure 2). In fact, the 2-back histograms of the simulated and the empirical data were very similar, with the exception of the return saccade peak. We thus conclude that the statistical structure of three consecutive saccades can be explained entirely from the statistical structure between pairs of saccades, with the exception of the increased amount of return saccades.

Despite the fact that the control trajectories do not preserve statistical effects of saccade triplets and saccade quadruples, we still compared the number of 3- and 4-back return saccades to the number computed from the control trajectories. In all cases, we observed many more return saccades in the empirical data (see Figure 3). We also found more return saccades than empirical forward saccades for 3- and 4-back saccades.

We conclude that locations that have been visited before are likely to be re-fixated, and for longer trajectories, this cannot be explained by the conditional dependencies between two consecutive saccades alone. We find that 1- to 4-back return saccades occur much more often than expected, but we do not observe any deviations from the predictions based on the statistics of saccade pairs for other saccades.

### Temporal Properties of Return Saccades

**Figure 4:**
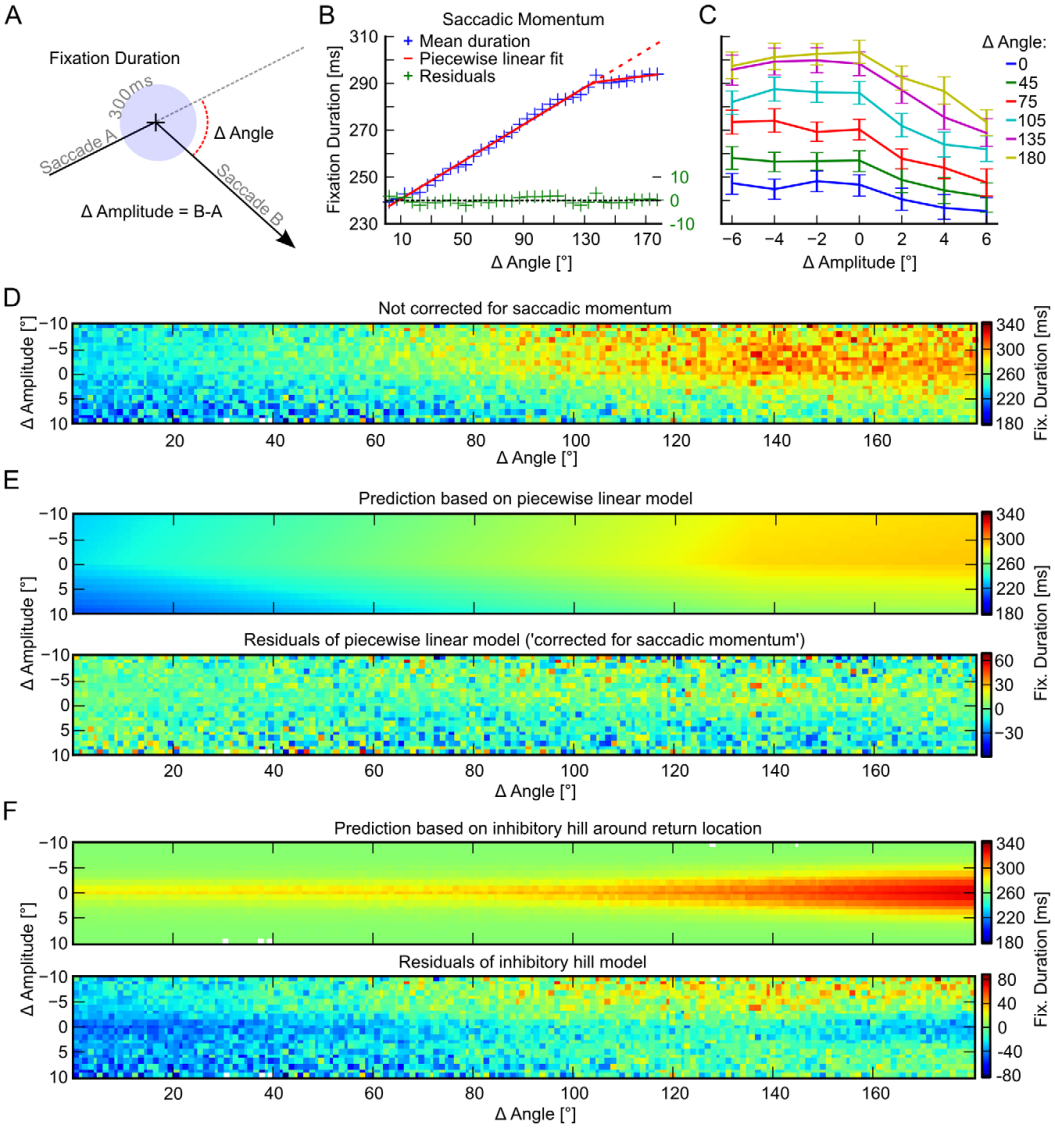
The saccadic momentum effect. A) Schematic drawing of plotted fixation durations, angle, and amplitude differences. B) Average fixation durations, corrected for the effect of saccade amplitude difference, as a function of the angle difference between two saccades (data is pooled over all subjects). Turning the direction of a saccade prolongs the fixation duration before the saccade is made. C) Shows average fixation durations for specific combinations of amplitude and angle differences (data is binned with bin sizes of 30° and 2° for angles and amplitudes respectively; errorbars are 95% CIs over subjects). This shows that there is no increase of fixation duration for return saccades, except for the effects of angle and amplitude differences. D) Same as C but with bin sizes of 1°; fixation durations are color-coded. E) Top panel: Prediction of average fixation duration based on the piecewise linear model (the fit is based on pooled data over all subjects for visualization purposes). Bottom panel: Residuals of correcting for angle and amplitude differences with the piecewise linear model. Here the fit was done for each subject individually, and we averaged after the correction. F) Top panel: Prediction of average fixation duration based on the inhibitory hill model (the fit is based on pooled data over all subjects for visualization purposes). Bottom panel: Residuals of the inhibitory hill model. Here the fit was done for each subject individually, and we averaged after the correction.

After investigating spatial properties of return saccades we turned to temporal properties. The investigation of temporal IOR is complicated by a dependence of fixation duration on the angle and amplitude difference between the incoming and the outgoing saccade (see Figure 4B and also [9]–[11]). On average, it takes longer to initiate a saccade perpendicular to the last saccade relative to a forward saccade, an effect termed ‘saccadic momentum’. Because this effect is reminiscent of classical IOR effects we wanted to explicitly account for saccadic momentum. To achieve this we fitted a piece-wise linear model to the fixation duration data of each subject. In a fixation sequence A→B→C the model predicted the fixation duration at location B based on the amplitude and angle differences between saccades from A→B and B→C. We used a piecewise linear model with two slopes for angle and amplitude differences respectively (Figure 4B,E). The slopes for angle differences changed at a critical angle that was fitted at the same time. However, the position of the slope change for amplitude differences was set to 0°. Please note that for visualization purposes Figure 4 shows models fitted on all data, but for the analysis models were fitted for each subject individually with a least squares procedure. The subject specific models accounted for 10% of the variance in the fixation duration data. Contrary to Smith and Henderson [9], we found that saccadic momentum did not increase linearly with the angle difference, but exhibited a change in slope for angle differences larger than 117° (CI [109, 124], slope of first segment 0.383 ms/°, CI [0.350, 0.416], and slope of second segment 0.002 ms/° CI [−0.13, 0.116], Figure 4A,B,E). The slope of the second segment is not significantly different from 0° and therefore indicates that no additional delay after the breakpoint at an angle difference of 117° occurs and that return saccades are faster than predicted by the first slope (see Figure 4B, compare red solid vs. dashed line). Thus, saccadic momentum is captured by a model with two different parts: up to angle differences of 117° fixation duration increases with 0.383 ms/° but larger angle differences do not incur a larger delay. We hypothesize that the different slopes might be due to two mechanisms that contribute to eliciting saccades with different dependencies on relative angle.

Amplitude differences changed slope at 0°, undershooting saccades had a slope of 0.39 ms/° CI [0.18, 0.60] and overshooting saccades had a slope of −2.75 ms/° CI [−3.02, −2.50].

In conclusion, the shallow slope for undershooting saccades, together with the position of the angle difference breakpoint at 117° and the 0 ms/° slope afterwards, show that the saccadic momentum effect is not specific to the return location.

Additionally we investigated IOR, similar to [9], by comparing over- and under-shooting saccades with an angle difference of 180±30°. Contrary to [9], we found no sign of a prolonging of exact return saccades (see Figure 4C, CIs for 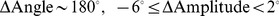 largely overlap) compared to undershooting saccades. To exclude a potential effect of binning, we repeated this analysis with bins that were only one degree wide (see Figure 4D).

We also wanted to rule out the possibility that, additional to the spatially unspecific saccadic momentum effect, a spatially specific temporal IOR effect existed. We therefore fitted an ‘inhibitory hill’ model to the data (Figure 4F). Similar to the piecewise linear model, in a triplet of fixations A→B→C, we predicted the duration of fixation B. But this time we assumed that a Gaussian like inhibitory hill centered on fixation A would increase the duration of fixation B. The size of the inhibition was proportional to the distance between fixations A and C. We fitted this model with a least squares procedure with inhibitory hills of different sizes. The best fitting model had a Gaussian inhibitory hill with 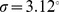 and explained 1.6% of the variance in the fixation durations. When we fitted the same model on the residuals of the piecewise linear model the variance explained dropped to less than 0.0001%. Hence, on its own the ‘inhibitory hill’ explains much less variance of the data than the piecewise linear fit and adding the ‘inhibitory hill’ model to the piecewise linear fit had virtually no benefit. We conclude that the residuals of the piecewise linear model do not contain an effect of temporal inhibition of return anymore.

Next, we investigated the effects of correcting for saccadic momentum with our piecewise linear model. Figure 4E (bottom panel and Material and Methods) shows the residuals of the piecewise linear model. We observe that fixation durations before return saccades are not systematically different from fixation durations before saccades to other locations (see Figure 4E bottom panel). In contrast, the residuals of the inhibitory hill model (Figure 4F bottom panel) show systematic dependencies on angle and amplitude differences between saccades.

In summary, the prolonging of fixation durations before return saccades can be explained in terms of saccadic momentum and saccadic momentum is not specific to return locations.

We next considered fixation durations at return locations to investigate if they are looked at more often because they were not scrutinized sufficiently the first time around [8] or because they are highly salient and also demand above-average processing time.

**Figure 5:**
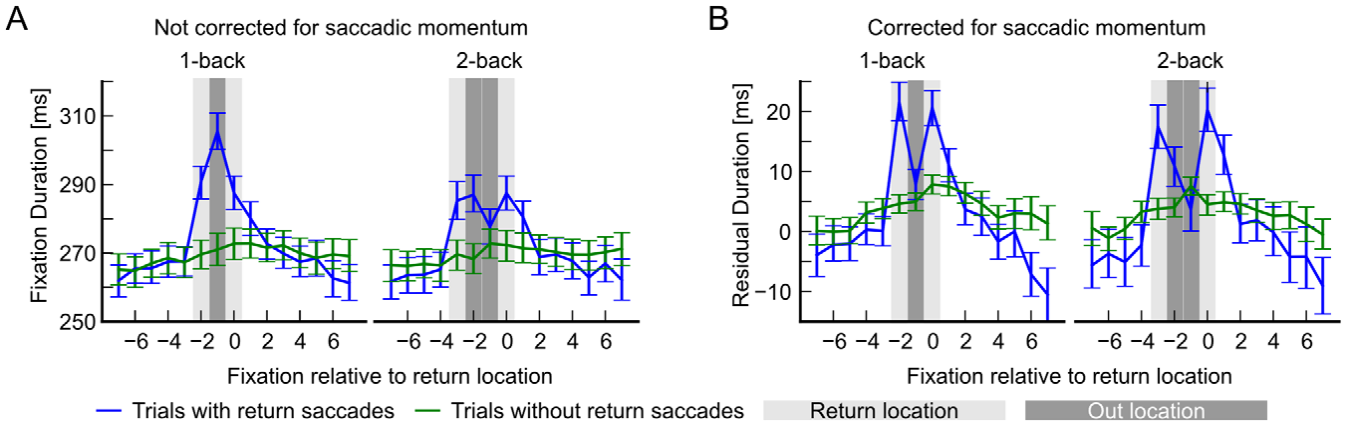
Fixation durations at return locations are longer. A) The average fixation duration at return locations in trials with 1-back and 2-back return saccades (blue lines) is longer than fixation durations in control trajectories (green lines). Errorbars are 95% CIs bootstrapped over subjects. B) Correcting for saccadic momentum with the piecewise linear model completely removes any trace of temporal inhibition of return for 1- and 2-back return saccades. Errorbars are 95% CIs bootstrapped over subjects.

To this end, we compared all trials (i.e. the entire fixation trajectory of one subject on one image) that contained return saccades (RS-trials) with all trials that contained no return saccade. We centered all RS-trials on the 2^nd^ fixation of the return location. We aligned trials of the same length without RS to the trials that contained a RS. If for example, the 2^nd^ fixation of the return location occurred at fixation Nr. 5, both trials were centered on fixation Nr. 5. Figure 5A shows that fixation durations at the return location are significantly longer than at control locations. Remarkably, this even holds when the location is visited for the first time. We observed the same pattern with a reduced effect size for 2-back return saccades. Hence, return saccades are not due to a shortened analysis at first fixation; fixation duration is significantly increased during first fixation and re-fixation.

Please note that correcting for saccadic momentum and saccade amplitude differences eliminates the increase in average fixation duration before the return movement, where IOR has been typically observed (see Figure 5B). This supports our conclusion that controlling for the effects of saccadic momentum explains the prolonging of fixation durations before return saccades in our data.

To check if saccadic IOR effects that could not be explained by saccadic momentum were present in the individual experiments that we analyzed, we repeated the comparison of RS-trials and non-RS trials for every dataset. We checked if the difference at the out-location between both trial types was significantly different from zero when we corrected for saccadic momentum with our piecewise-linear model. We did not find any significant deviations (paired T-test, 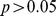, Bonferroni corrected).

In summary, in our data temporal effects of IOR can be accounted for by a pronounced, non-linear effect of saccadic momentum and saccade amplitude differences, which is not specific to the return location. Additionally, the average fixation duration at the return location is longer for 1- and 2-back saccades, already during the first visit.

### Return Saccades and Saliency

The observation of increased fixation duration at return locations suggests that such locations are special. To investigate whether the stimulus was systematically different at return locations compared to regular fixations, we computed bottom-up saliency at both locations based on the values of a large number of low (e.g. luminance, red-green and blue-yellow contrast) and mid-level (e.g. symmetry, intrinsic dimensionality) stimulus features.

**Figure 6:**
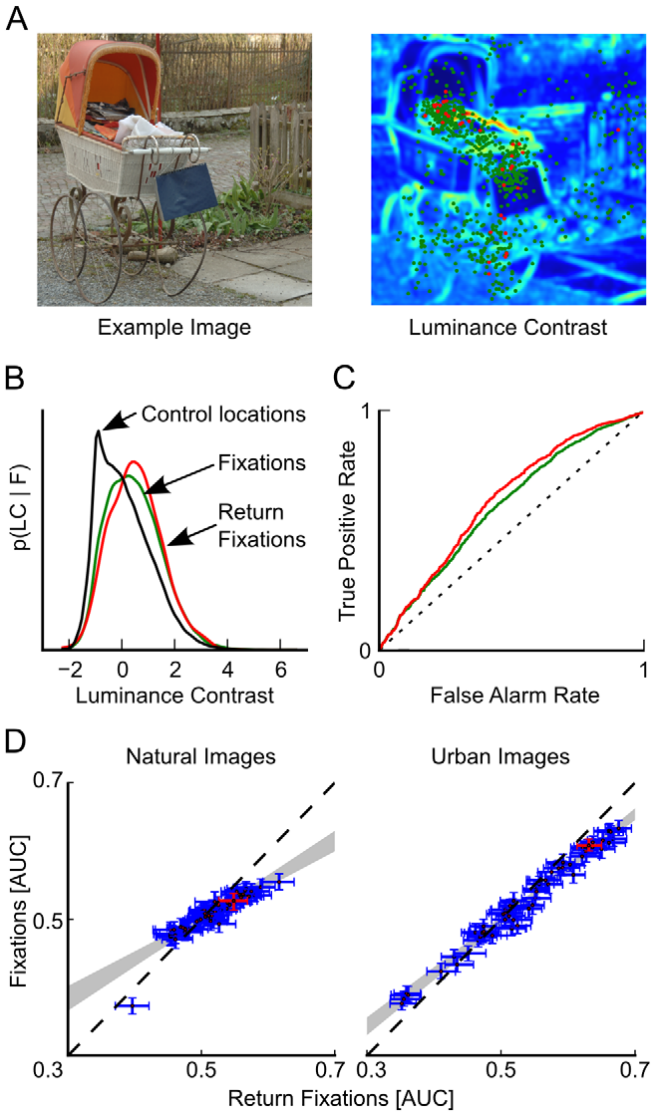
Image features predict return locations better than normal fixation locations. A) The right panel shows the luminance contrast feature for the image on the left. Green dots mark regular fixations, and red dots mark return locations. B) The distribution of feature values at control locations, regular fixation, and return fixation locations. C) The ROC curve for separating regular and normal fixations from control locations. D) AUC values of individual image features for return and regular fixation locations. Return locations are systematically better predicted by image features than regular fixations—i.e., return location feature AUCs are higher for predictive features (AUC>.5) and smaller for anti-predictive features (AUC<.5). Error bars are bootstrapped 95% CIs. The relationship between regular feature-fixation AUCs and return feature-fixation AUCs is well described by a linear relationship 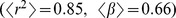. Gray shaded area: convex hull of regression fits between return and regular feature AUC patterns.

We compared the values of 63 local features (please see Materials and Methods: Feature Analysis for the complete list) at return and non-return (normal) fixations in the dataset used in [17] (Figure 6A,B). For quantification, we computed the area under the receiver-operating characteristics curve (AUC) of a linear classifier that separates return and normal fixation locations from control locations on the same image (Figure 6C) [18]. The AUC measures how well a feature can be used for correct classification. 0.0 implies perfect classification but switched labels; 0.5 is chance performance; 1.0 is perfect. Control locations were sampled from all fixation locations made on other images from the one in question and hence take into account the general spatial bias. We calculated the AUC for separating return fixation locations from controls and the AUC for separating normal fixation locations from controls.

We observe a linear relationship between AUCs of different features calculated for return-locations and normal-fixations. Furthermore, this holds for natural and urban scenes (Figure 6D, each data point shows AUC values for one image feature). The pattern of AUC values for return and normal fixations is well described by a linear relationship (natural scenes: 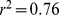, urban scenes: 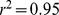). Only the phase congruency feature does not fit this linear pattern; it is slightly better for predicting normal fixations than return fixations (lower left corner in left panel of Figure 6 D). Importantly, the slope of the linear fit is less than 1.0 (natural scenes: 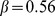, T-test 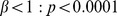; urban scenes: 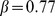, T-test 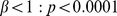). Hence, those features that predict normal fixation locations above chance (AUC>.5) better predict return locations than regular fixation locations. Importantly, those features that are anti-predictive (AUC<0.5) are also more anti-predictive of return locations than of regular fixation locations. This indicates that the pattern of contribution of different features, as quantified by the AUC values, does not differ between normal and return locations. Such a linear relationship implies that image feature based salience models trained only on regular fixation locations will perform better on return locations than on regular locations. In summary, image features better predict return locations than regular locations.

To compare bottom-up saliency values at return and normal fixation locations, we compiled a weighted sum of all 63 features into a single saliency score. Weights for the linear combination were obtained by a logistic regression that separated either return locations from controls (RS-model) or normal fixations from controls (FIX-model, see Materials and Methods). We then computed the AUC of both saliency scores for separating return-locations and non-return locations from controls. We used leave-one-subject-out cross validation to ensure independence of training and test data. We found that return locations could be better predicted (average AUC of 0.733; RS-model: 0.731, FIX-model: 0.736) compared to normal fixations (average AUC of 0.670; RS-model: 0.667, FIX-model: 0.674). From this analysis, we conclude that return saccades are directed to more salient locations than normal saccades and that the pattern of feature-fixation correlations is comparable to return locations and normal fixations.

### Fixation Sampling Strategies

**Figure 7:**
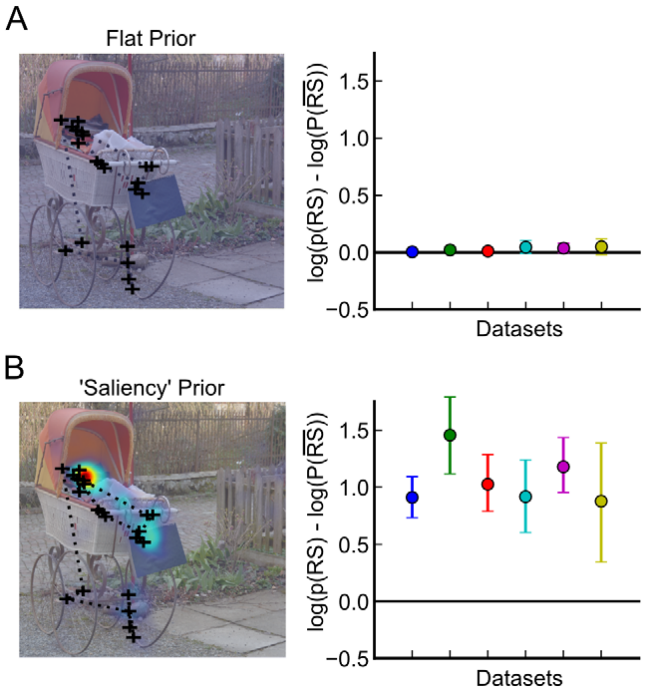
Return saccades increase the trajectory likelihood when observers sample from an empirical saliency distribution. Both plots show the difference of the log likelihood for trajectories with and without return saccades as a function of the dataset. A) If all locations have equal probability of fixation, trajectories with return saccades are as probable as trajectories without return saccades. B) If salient locations are more probable than other locations, trajectories with return saccades are more likely than others. Error bars are bootstrapped 95% CIs.

The finding of an increased number of return saccades and prolonged fixation durations at return locations is difficult to reconcile with a foraging strategy that maximizes the entropy of a fixation density map, i.e. the area that is ‘covered’ by fixations. Yet return locations are ‘special’ in the sense that they are looked at longer and do not appear at random locations. Instead of maximizing entropy, we hypothesize that the very existence of return fixations serves to optimize the match of saccadic trajectories with an internal priority map that encodes which locations are relevant in the scene. Here we replace the spatially flat prior of the maximal entropy assumption (Figure 7A) with a stimulus-dependent prior and use the viewing behavior of (other) subjects as a proxy for such an internal priority map (Figure 7B). That is, we use empirically defined salience, given by how often different subjects look at a location, as the internal priority map.

In this respect, we were interested if, all else being equal, a return saccade would increase the probability of a trajectory according to the internal priority map. We compared trajectories with return saccades to the same trajectories that, instead of exploiting an already seen location, explored one additional new location.

More specifically, for each fixation trajectory that contained a return saccade, we first computed a fixation density map from the fixations of all other subjects on the same image. We made sure that in this computation, trials containing return saccades were omitted (see Materials and Methods). We then used this fixation density map as an internal priority map for the trial in question. We compared the probabilities of generating two different kinds of trajectories based on the fixation trajectory in question from this internal priority map: The first contained the return saccade (return-trajectory) but we removed the last fixation. For the second trajectory (exploration-trajectory) we removed the 2^nd^ visit to the return location but kept the last fixation. The exploration and return trajectories thus contained the same number of fixations, but the exploration trajectory contained one more unique fixation location (see also Materials and Methods). In other words, given the original fixation trajectory A-B-A-C … -F-G, the return trajectory is given by A-B-A-C…F and the exploration-trajectory is A-B-C…-F-G.

The probability for the exploration and return-trajectories was defined as the probability to draw exactly these trajectories from a multinomial distribution with event probabilities given by the internal priority map. Because we use a multinomial distribution as our model, the order of fixations is irrelevant and changed distances between fixations do not confound the results. We find that return saccades actually increase the probability of a trajectory compared to the omission of such saccades (Figure 7, ANOVA with factors experiment and saliency map type, main effect of saliency map type 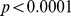, no other significant effects at 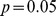).

In summary, to match an internal priority map it is better to allow return saccades to exploit empirically salient locations in the priority map compared to forcing all saccades to unexplored locations. This result is also reflected in the additional finding that return locations show higher average values of the internal priority map compared to locations before and after return locations. That is, humans try to visit empirically highly salient regions but trade off exploitation and exploration by revisiting important parts of the stimulus.

## Discussion

In this study, we investigated the spatial, temporal and functional properties of saccadic inhibition of return.

With respect to spatial properties, we find more 1-back and 2-back return saccades than expected from the distribution of saccade angles and amplitudes and relative angles and amplitudes. Also, our novel statistical model for 2-back return saccades reproduces the distribution of angle and amplitude differences of saccade triplets very well except for 2-back return saccades. This indicates that our model is adequate to explain higher order biases in saccade trajectories but that 2-back return saccades are facilitated compared to these higher order biases.

This agrees with findings from Hooge, Over, van Wezel and Frens [8], who used a comparable baseline for 1-back return saccades. Smith & Henderson [9]–[11] used two different baselines but find similar results. Compared to distance matched controls (e.g. saccades with 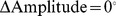 and 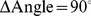) they report an equal or larger number of 1-back and 2-back return saccades. Compared to a baseline where the order of fixations is shuffled they report more 1-back and 2-back return saccades in their empirical data.

In disagreement with our results Bays and Husain [19] argue that 1-back return fixations occur less often than should be expected. The critical difference to our study is the baseline used for comparing the number of return saccades. Bays & Husain argue that saccade trajectories are not only influenced by oculomotor biases but also by the spatial distribution of salient locations in an image. They generate control trajectories that take both biases into account by sampling from the conditional probability distribution 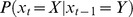, which expresses the probability to fixate location X given that the current fixation is at location Y. Importantly, the resulting trajectories contain more return saccades than their empirical data. Because the process that generated these trajectories did not take into account past fixations but still created more return fixations, Bays and Husain conclude return locations are actively inhibited. What could explain the differences between our and Bays & Husain's findings? There are several differences regarding the acquisition and analysis of eye-tracking data in our and Bays and Husain's study. First, Bays and Husain presented images for 20 s while in the present investigation the presentation time was 6 s or shorter. Fixation trajectories over repeated presentations of the same stimulus are partly overlapping [16], supporting the argument that prolonged stimulus presentations might have an effect on the frequency of return-saccades. Second, we seem to observe a much more localized return peak (compare our Figure 2B and their Figure 1B). This percolates to differences in the definition of return saccades between Bays & Husain's and our work and thereby to a different estimate of the number of empirical return saccades. Third, to accurately estimate conditional probability densities a large amount of data is required. In the present study we opted for a reanalysis of several previously conducted studies resulting in a very large database. This allowed us to remove trajectories containing return saccades from the estimate of fixation probability densities. Fourth, typical laboratory setups with limited size monitors enforce saccades with larger than 90° turning angle in order to maintain the gaze within the monitor boundaries, part of these are classified as return saccades. To resolve these issues and reach a final conclusion more research is warranted.

With respect to the temporal properties of return saccades, we find that direct return saccades are preceded by longer fixations than forward or perpendicular saccades. We therefore replicate a classical effect of saccadic inhibition of return. However, this effect is explained by saccadic momentum [9], [13]: A piecewise-linear dependence of fixation durations on angle and amplitude differences between the preceding and next saccade, i.e. large turns in eye movement direction are preceded by longer fixation durations compared to small turns. We find that exact return saccades do not take longer than undershooting return saccades, or saccades with an angle difference larger than ∼117°. Correcting for the effect of saccadic momentum removes the delay imposed on direct return saccades compared to non-return saccades. Crucially, we find that the dependence of fixation durations is constant after a critical angle. That is, return saccades are faster than expected from the slope of saccadic momentum before the critical angle. We furthermore tested directly if a localized ‘inhibitory hill’ around the return location could explain our data and found that it performed worse than the piecewise linear model. Additionally, the inhibitory hill model did not explain variance in our data that was not explained by the piecewise linear model. In conclusion, we did not find a spatially localized inhibitory effect for return saccades in addition to saccadic momentum. We therefore conclude that our data is better described by a spatial facilitation of return, a delay increasing linearly with angle of changing gaze direction (saccadic momentum) up to a critical angle and constant thereafter.

Our results are compatible with many findings in the literature. We replicate the classical saccadic IOR effect [2], [8] on a large data set and provide a detailed description of saccadic momentum [9]–[11], [13]. However, we could not replicate Smith & Henderson's [9] finding of an extra delay, in addition to saccadic momentum, for return saccades. Also, our results contrast with findings of Hooge and Frens [12] who find a localized temporal zone of inhibition of 4° by asking their subjects to carry out a pre-determined sequence of saccades. The difference between Hooge et al.'s and our results might be explained by the two very different tasks and stimulus arrangements. It is well known that the oculomotor system in the brain includes many different areas that are tightly coupled [20]. Carrying out pre-programmed saccade sequences might recruit neural substrate that elicits temporal inhibition of return. Hooge et al. suggest that the superior colliculus might be the neural substrate that causes effects observed in pre-planned saccade sequences. In contrast, free viewing, where fixation locations are selected based on local salience, oculomotor bias's and other top-down factors, might activate different networks that lead to different temporal properties of fixations. One candidate would be LIP which has been implicated in computing a priority map [21] which combines bottom-up and top-down information to guide selection of fixation targets during visual search.

An alternative non-exclusive explanation, that would incorporate both contradicting results, might be that precise saccadic IOR can be tuned by the visual system. This is supported by a study from Farell, Ludwig, Ellis, and Gilchrist [22] that shows that the classical IOR effect is adaptive to environmental statistics. However, because they did not explicitly investigate saccadic momentum, it remains to be seen what is modulated: a return location unspecific saccadic momentum, return location specific IOR or both.

Interestingly we find that return locations are more salient, according to empirically measured as well as stimulus dependent saliency, than regular fixations. Hooge et al. [8] find that the first visit of a return location is shorter than the second visit. They suggest that return saccades occur because the visual system did not have enough time to analyze a fixation location during the first visit. In our data return locations are fixated longer compared to control fixation locations during both visits. The visual system therefore has more processing time available for return locations than for regular locations. These findings suggest that return locations need to be scrutinized in more detail than regular fixation locations.

We also found that return saccades increase the match of individual trajectories with a grand total priority map. The priority map was defined by empirical salience, i.e. those locations that are consistently fixated by many subjects. Because trajectories that contained return saccades were more likely than trajectories that explored a new location with every fixation, we suggest return saccades are the consequence of a fixation selection strategy that samples relevant parts of a scene. Furthermore, because the internal priority map was defined by empirical salience, which we interpret as a proxy for behavioral relevance, return locations were more relevant than other locations. We therefore suggest that the fixation selection strategy trades off exploration of unseen relevant locations and exploitation of already seen relevant locations with return saccades.

### What Are Possible Mechanisms that Could Explain Our Findings?

With respect to saccadic momentum, the question arises whether the observed regularities could be an effect of the physical properties of eye-movement control. Different patterns of muscle movements are necessary for return saccades and forward saccades. Forward saccades require flexed muscles to be flexed more, while stretched muscles must be stretched more. Return saccades require an inversion of these muscle states: flexed muscles must be stretched and stretched muscles must be flexed. This might contribute to the observed differences in fixation durations. However, when talking about muscle effects, two things should be kept in mind: First, the temporal difference between the length of fixations before return and forward saccades is in the order of 50 ms (Figure 4B). Bahill, Clark and Stark [23] show that saccades of up to 20° can be completed in less than 60 ms and thus it is safe to assume that the time needed for the acceleration of the eye during a saccade is much shorter than 50 ms. Therefore, differences in activation patterns on the muscular level cannot explain the systematic increase of fixation durations observed here.

Second, Farell, Ludwig, Ellis, and Gilchrist [22] found that the temporal difference between return and forward saccades is modulated by the likelihood of a return saccade, with the effect eventually vanishing when return saccades are very likely. While this does not rule out a contribution of muscle effects to saccadic momentum, the least it demonstrates is that saccadic momentum can be modulated by factors that are independent of physical motor control.

Ludwig, Farell, Ellis, and Gilchrist [24] proposed that ‘Inhibition of Return’ can be explained in a decision-making framework. In short, potential saccade targets accumulate evidence until an evidence threshold is reached. The first target that reaches the threshold is used as the next saccade target. Indeed, they find a difference in saccade latency for return and non-return saccades that correspond to differences of accumulation rate in their fitted models. However, they only differentiate return and non-return saccades. Thus, the phenomenon of saccadic momentum makes a large contribution to that comparison and might easily dominate. It would be interesting to see if the accumulation rate is parametrically modified by the angle difference between the new saccade target and the last saccade. However, even if a change in accumulation rate can explain saccadic momentum, this would make the high incidence of return saccades even more puzzling. Furthermore, the question remains why the accumulation rate changes with changes in eye-movement direction.

But we find an alternative suggestion more tenable: If we imagine that we shift the center of gaze from point A to B, then parts of the stimulus between A and B will have been sampled by the fixation of A. Thus, relative to B, backward targets are at locations for which prior information exists while forward targets deal with parts of the stimulus for which no (or less) information is available at that moment in time. We hypothesize that forward and backward targets have different accumulation rates because different amounts of knowledge are available for these locations. Considering that receptive fields are remapped during saccades, it does not seem unlikely that prior knowledge is transferred during the remapping [25]. For salient targets to reach the decision threshold faster when they are ‘forward’ compared to ‘backwards’, the accumulation rate has to be inversely proportional to the amount of knowledge available. This would imply that more prior knowledge leads to slower accumulation of evidence. It seems that such a notion is compatible with accounts of predictive coding in which higher-level information explains away activity at lower levels [26]. Here the higher-level knowledge about backwards locations ‘explains away’ their salient properties, thereby making them less salient compared to forward locations. This in turn would lead to a slower accumulation of evidence at backward compared to forward locations. In that context, the observed piecewise linear dependence of fixation duration on saccadic angle is important. Based on this observation we suggest that two competing mechanisms are active in parallel, and only one of these—the one with a steep dependence of fixation duration on the saccadic angle—is dependent on the already available knowledge. While this is speculative, the proposal does fit with our finding of increased bottom up saliency at return locations.

In summary, accumulator models are a promising tool to understand the dynamics of saccade target selection. Future studies will need to link saccadic momentum and facilitation of return to specific properties of such models. The findings that return locations are more salient and looked at longer must be crucial parts of this puzzle.

### What Could Be the Function of Facilitation of Return and Delay of Direction Change?

Clearly, spatial facilitation of return is incompatible with the objective of covering the entire stimulus evenly with fixations in a short amount of time. However, what is the motivation to assume that the stimulus is equally interesting in all locations? In an everyday search task, such as when looking for the car keys, one would not cover all places from cellar to rooftop evenly. Instead, it is sensible to scrutinize those locations that are likely due to prior knowledge and to look twice before considering more exotic alternatives. Under laboratory conditions, for example when a near threshold Gabor patch is superimposed on a pink noise image at a random location, the search strategy might adapt to the flat location prior [27]. This is a remarkable feat of behavioral adaptation, yet no reason to assume that return saccades are generally inhibited. During free viewing, no explicit external task is enforced and subjects do not relate their eye-movement behavior to an externally set optimality criterion. Some studies included in our data set employ specific tasks. Specifically, in the delayed patch recognition task [14], subjects have to decide whether a target patch was contained in a previously shown sample image. The target patches are selected uniformly from the entire stimulus, which might suggest that return saccades are not useful to solve the task. However, the probe patch is not presented in the location where it was in the stimulus and after stimulus offset only. This makes keeping track of where in the sample image the target patch was selected difficult. Furthermore, to prevent fatigue, [14] deliberately choose to present only 128 images to each subject. The number of trials is therefore considerably smaller than in psychophysical studies with reduced setups. Hence, the opportunity for subjects to infer and adapt to the objective prior of patch locations is rather limited. But even for fully adapted subjects, it is unclear whether seeing the entire stimulus is the optimal strategy for a delayed patch recognition task. The task requires not only passive observation of the stimulus but encoding and recalling as much as possible of it at a later stage. The optimal strategy needs to trade off holding complex stimulus patches in memory with exploring new parts of the stimulus. In this respect, return saccades might be part of an optimal strategy because they allow the visual system to exploit information at relevant locations more thoroughly.

It could be argued that we did not use a visual search task and therefore found more return saccades than expected. As described above two studies included in our data set employed a delayed template match search task where homogeneously distributed fixation locations seem advantageous. Furthermore, even during visual search return saccades are not automatically disadvantageous for search performance. First, a consistent central bias has been documented in many studies (for example [17], [28]), invalidating an assumption of a flat prior. This shows that the visual system does not consider every location to be equally relevant. Second, Hooge, Over, van Wezel and Frens [8] find more 1-back return saccades than expected during a visual search task. Third, even during visual search, a single fixation might not suffice to identify a target in front of the background, and there is evidence that humans take uncertainty inherent in their visual system into account [27]. Also, there clearly are prior expectations about where targets of specific types can be found in a scene [29] (e.g., pedestrians are usually not located in the sky). These two conditions necessitate trade off of exploration and exploitation in visual search—return saccades (exploitation) with saccades that target unseen parts of the stimulus (exploration). Therefore return saccades are likely to be a part of visual search strategies as well.

Having considered everyday search tasks, free viewing, and delayed patch recognition, we find it unconvincing that a flat spatial prior over stimulus locations must be part of a good strategy to solve these tasks. In turn, we argue that from the existence of return saccades, it does not follow that a task is not being solved optimally.

Instead, a novel view concerning the functional interpretation of IOR emerges. Farell et al. [22] have shown that the time difference between return and forward saccades is adaptive to the environment. Smith & Henderson [10], [11] argue that saccade latencies are the result of several interacting processes such as bottom-up input, top-down control and saccadic momentum. We provide evidence for the hypothesis that return saccades are part of a strategy that aims at devoting attention to the most relevant information in the stimulus: First, return locations are more bottom-up salient than regular fixation locations, showing that the stimulus is different at return locations compared to regular locations. Second, return locations are fixated longer during both visits, indicating that more attention compared to regular fixation locations is devoted to return locations. Third, return saccades occur more often than expected, suggesting that they are an important part of a fixation selection strategy. Most importantly, if we accept eye movement behavior of other subjects as a proxy for relevance, return saccades increase the likelihood of a trajectory to sample the relevant parts of an image. We therefore conclude that spatial facilitation of return, saccadic momentum, and relative speed-up of saccades at very large angle differences might not serve a single objective but might emerge from the broader goal to optimally sample relevant parts of a stimulus.

## Materials and Methods

### Data

We re-analyzed data from several studies conducted at the Institute of Cognitive Science, University of Osnabrück. Here we briefly summarize the different studies but leave details to the respective original publications. Açik et al. [14] investigated the effect of age on viewing behavior. They presented 128 images from the categories ‘manmade scenes’, ‘natural scenes’, ‘fractals’, and ‘pink noise’ for 5 s. The images were selected from a larger database that contained 64 images per category. Images from the same database were used in [15], [17]. After each image, an image patch was presented, and subjects had to answer whether this patch was contained in the previously shown image. Fifty-eight subjects participated in this study (18 elementary school children with a mean age 7.6, 23 university students with a mean age of 22.1, and 17 older adults with a mean age of 80.5). Wilming et al. [17] showed 128 images from the categories ‘manmade scenes’ and ‘natural scenes’ in a free viewing paradigm with a viewing duration of 6 s to 48 subjects (aged 19 to 28 years). Kaspar and König [15] investigated the influence of repeated stimulus presentations, image category, and individual motivations. They presented 48 images taken from the same scene types used by [14] and repeated the presentation of each image 5 times. The subjects were instructed to freely view the image for a period of 6 s. Forty-five subjects participated in the study (aged 18–48 with a mean age of 24.2 years). Kaspar and König [16] (data from ‘Experiment 2’) presented 30 different urban scenes to 34 subjects (aged 19–49, mean age 25.9 years) with a viewing duration of 6 s. Each image was presented five times to each subject. The images were not part of any of the other studies used here. We analyzed data from two more experiments; the results have so far not been published. In these studies, conducted by Alper Açik, 50 subjects were presented with contrast modified and phase scrambled images from the category ‘fractals’. After a stimulus presentation of 5 s, subjects had to perform a 2AFC patch recognition task (20 subjects) or a YES/NO patch recognition task (30 subjects). We treated the two different tasks as different datasets.

All studies used an Eyelink II eye-tracker (SR Research Ltd., Mississauga, Ontario, Canada). All studies were conducted in compliance with the Declaration of Helsinki as well as national and institutional guidelines for experiments with human subjects. Because different studies used different displays and image sizes, we converted all fixation coordinates into degrees of visual angle. In total we analyzed over 597,000 fixations collected from 235 subjects in 6 different studies.

### Spatial Properties of Trajectories

To investigate the frequency of 1- and 2-back return saccades, we created two different baseline conditions. For the 1-back condition, we shuffled all of the recorded saccades. This removed all order effects but did not change the distribution of saccade angles and amplitudes. We used this shuffled baseline to estimate how many return saccades should be expected by randomly sampling from the distribution of saccade angles and amplitudes. All saccades with an angle difference larger than 178° and amplitude difference of less than ±2° were considered return saccades. To determine significant deviations of the number of return saccades from the shuffled baseline, we bootstrapped 95% confidence intervals around the mean difference of return saccades for each subject and checked if the confidence interval contained 0. In comparison, the empirical data contained significantly more return saccades in the 1-back condition. Bootstrapping the per-subject percentages created the 95% confidence intervals shown in Figure 3.

Subsequently, to investigate whether 2-back and higher dependencies between saccades can be explained by 1-back information, we devised a saccade generator, which uses 1-back information of trajectories as an input to generate arbitrarily long sequences of saccades. As the generator does not use any 2-back information, any patterns that can be observed in the 2-back condition of the generated data are due to 1-back dependencies alone. The generator creates a trajectory by drawing a saccade from the distribution of first saccades in the input data and copies its absolute angle and amplitude. Subsequently, further saccades are added by drawing their angle difference and amplitude with respect to the last saccade from the conditional distribution 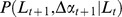. This distribution expresses the probability of observing an amplitude difference 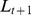 and angle difference 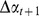 at the next saccade, given that the length of the last saccades was 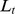. It thus comprises only 1-back information. We estimated this distribution for every subject separately by computing histograms for each possible value of 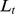. Sampling from the non-conditional probability distribution from Figure 2 does not generate valid adjoining saccade trajectories because not all negative amplitude differences can be generated at all times. In terms of fixation coordinates, no additional restrictions were made such that the simulator precisely replicates 1-back dependencies without incorporating any additional image statistics such as picture size. The resulting set of fixations could be analyzed in terms of saccade dependencies equal to the empirical data. To validate the accuracy of the saccade simulator, we compared the similarity between subjects and the similarity between subjects and simulator. To this end, we computed for each subject the distribution 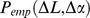 of amplitude differences 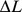 and angle differences 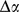 (see Figure 2) for 1-back saccades. Subsequently we computed 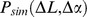 for each subject based on saccades generated from their own distribution 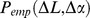. Finally we computed the KL-divergence between subjects and between subjects and their simulated saccades:
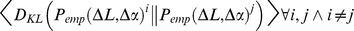


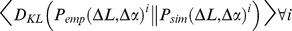
where i and j are subject indices. We found that the KL-divergence between subjects was higher than the divergence between subjects and simulator output. Additionally, the number of return saccades generated by the simulator is not different from the number of return saccades found in the empirical data. Furthermore, qualitative comparison of differences between empirical and simulated data did not reveal any systematic deviations in the 1-back case. From this, we conclude that the simulator reliably replicates all 1-back dependencies in the data.

To compare the number of return saccades, we again bootstrapped 95% confidence intervals around the difference of simulated and empirical return saccades and checked if the interval contained 0. As expected, this was the case for 1-back saccades. All other comparisons showed significantly more empirical return saccades (see Figure 3). In the case of forward saccades, all comparisons contained 0.

To assess the similarity of the distributions 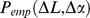 and 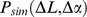 for the 2-back case (see Figure 2, left column), we calculated the KL-divergence between the two for each subject. The mean KL-divergence was 0.21, to which the return peak contributed more than any other area of comparable size (for example, 4 times as much as the forward peak). Thus, all other 2-back dependencies were very similar.

### Temporal Properties of Return Saccades

Because effects of saccadic momentum on fixation durations are largest at return locations, they potentially confound findings of IOR. [9], [11] considered the effect of saccadic momentum by comparing average fixation durations for exact return saccades and over- and under-shooting return saccades. We repeated this analysis but take several other measures to ensure a fair comparison. First, we explicitly estimated the effect of saccadic momentum and saccade amplitude differences on fixation duration with a non-linear breakpoint regression:
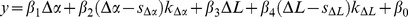
where 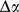 is the angle between the previous and next saccade, 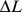 is the amplitude difference, 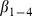 are the slopes of the individual linear segments, 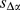 is the critical angle, 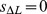 and 
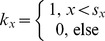
. The parameters 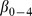 and 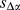 were fitted with a least squares procedure implemented in SciPy 0.9 for each subject individually. Please note that, for visualization purposes, the model fit in Figure 4 was computed by using all of the available data. All other inferences are based on models that were fit on a per-subject basis. We chose a piecewise-linear regression for two reasons: First, the relationship of angle differences and fixation durations seem to exhibit two linear parts (see Figure 4B,D). That is, using a linear regression introduces systematically larger residuals for large angle differences and for small amplitude differences. This is potentially critical because according to our data, changes in slope are not specific to return locations, and thus do not represent a true IOR effect but instead might interact with inferences about effects of IOR. Second, the breakpoint regression is conceptually simple and provides a decent fit with the data (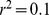, normalized RMSE = 0.16). Analyses that are ‘corrected for the saccadic momentum effect’ are carried out on the residuals of this regression.

Consecutively, we computed the duration of fixations with respect to the amplitude difference of the previous and consecutive saccade. Figure 4C shows an average over subjects for 30° bins for different saccade amplitude differences. Confidence intervals are based on bootstrapped across subject averages. Figure 4D shows the same but pooled over all subjects and for 1° bins for both angle and amplitude differences. Figure 4E shows the residuals of our piecewise-linear model, that is fixation durations corrected for saccadic momentum. Qualitative inspection shows that little structure remains in the residuals. Specifically, those areas where few samples are available (compare with Figure 2B, top left panel) show larger deviations than those where many samples are available. In an additional analysis (see Text S1) we found that such deviations can be expected even if no effect of angle and amplitude differences is present in the residuals.

Figure 5 shows trials with return saccades aligned to the return fixation and trials without return saccades. Trials without return locations were aligned as follows: For every subject we estimated 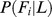 which expresses the probability that a fixation at location 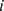 within a trajectory is a return fixation given that the amplitude of the trajectory is 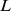. For every non-return trial we drew a return fixation location from this distribution and aligned the trial to this position. Error bars show bootstrapped 95% confidence intervals.

### Feature Analysis

To assess the relationship of return locations and bottom-up saliency, we used a saliency model similar to [30]. We computed 63 different features that are predictors of fixation locations on plain RGB values of the images. We used luminance, saturation, blue/yellow color, and red/green color channels of the stimulus [3]. All features were computed on three different spatial scales, which were created by rescaling the input image with a Gaussian pyramid. For each feature on each spatial scale, we applied three different filters: Gaussian smoothing 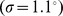, local contrast 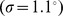, and texture contrast by calculating the local contrast twice on a feature 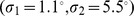. The local contrast is computed by 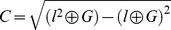, where 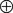 is the convolution operator and G is a Gaussian kernel with 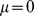 and 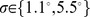.

Additionally, we computed intrinsic dimensionality [31], ID0, ID1, ID2, each with three different kernel sizes (0.12°, 0.52°, 1°), phase-congruency, and phase-symmetry [32], [33] as features. We furthermore considered several interactions of these features. We subtracted red/green contrast, blue/yellow contrast, saturation, and saturation contrast (all finest spatial scale) from phase-congruency and symmetry. Concerning intrinsic dimensionality, we compute ID0_0.25°_– ID0_1°_, ID0_1°_ – ID2_0.52°_, ID2_1°_ – red/green, ID2_1°_-saturation, ID2_0.12°_ – phase congruency. Together with the two last interactions, red-green contrast - saturation contrast and luminance contrast - saturation contrast, this yields 63 different features. Each feature map for each image was z-scored before it was used for further analysis.

To quantify how well a feature can predict fixations and return locations, we used the area under the receiver-operating characteristics curve (AUC). In short, the AUC assesses how well fixations can be separated from control locations on the same image based on the value of a feature at those locations [17], [18]. For every feature, we computed the AUC for separating normal fixation locations from control locations and the AUC for separating return locations from control locations. Control locations were chosen from the distribution of fixations on other images, which ensured that control locations follow the spatial distribution of fixations but were not actually fixated locations. We estimated the variability in the data by repeatedly (N = 150) computing both AUCs based on 1000 randomly sampled fixation and control locations. Confidence intervals were subsequently bootstrapped (N = 2000) on these 150 AUC values for each feature. The dependence between patterns of AUC values was well described by a linear relationship (natural scenes: 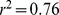, urban scenes: 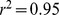). Figure 6D shows the AUC value of every feature for urban and natural scenes with bootstrapped CIs.

To further investigate the relationship between saliency and return locations, we assigned a saliency score to fixations and return locations. A saliency score was obtained by optimally combining features linearly to separate fixations (or return locations) from control locations. The weights for this combination were estimated with a logistic regression that tried to separate fixations from control locations based upon the 63 features. We used feature values at fixated locations as positive samples and feature values at control locations that were fixated on other-images as negative samples for the logistic regression. To test the hypothesis that return locations are more salient than normal fixations, we estimated two-saliency models and assessed how well return-locations can be predicted in comparison to normal fixations. The two models differ with respect to the samples used for training. The return-saccade (RS) model uses only return locations as positive samples, while the fixation (FIX) model uses only fixation locations from trials where no return saccade occurred. Both models were trained repeatedly by splitting the available data into test and training sets. We used leave-one-out cross-validation, where each subject was used for testing once and was not used for training in this run, this ensured that training and test data was completely independent. Both models predicted return locations and normal fixations separately. We found that return locations could be predicted with an average AUC of 0.73 (RS: 0.724, FIX: 0.731) compared to an AUC of 0.67 (RS: 0.667, FIX: 0.674) for normal fixations. A two-way analysis of variance with factors ‘model type’ and ‘fixation type’ revealed that both main effects and the interaction between the two are significant (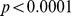).

### Fixation Sampling Strategies

To compute an internal priority map for a given subject and image, we computed a 2D histogram of fixation locations of all other subjects that did not make a return saccade on the same image from the same dataset. To obtain a density map, we convolved this histogram with a Gaussian kernel with full-width-half-maximum = 1° and normalized the filtered histogram to unit area.

To evaluate the likelihood that a trajectory is drawn from an internal priority map, we interpreted the internal priority map as cell probabilities for a multinomial distribution. How often a location is fixated gives the counts for each cell. The probability of a trajectory is then given by
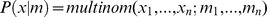
where 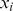 encodes the number of fixations for location 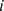, and 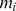 is the probability of the internal priority map at location 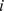. Subsequently, we compared two different trajectories. In one, the return location is fixated twice, but the last fixation is omitted. In another, the return location is fixated only once, but the last fixation is not omitted. These trajectories differ only in how often the return location and the last fixation are fixated. Thus, the entire comparison amounts to a comparison of internal priority map values at the return location and the last fixation of the trajectory. However, 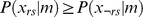 is only fulfilled when the priority map value for the return saccade is at least twice as large as the value for the last fixation.

## Supporting Information

Figure S1 Confidence intervals for the hypothesis that no angle and amplitude effect is present in the residuals of the piecewise-linear model. A shows the upper 97.5% confidence boundary as a function of amplitude and angle differences. Values are larger where fewer samples are available. B shows the percentile of the residuals of the piecewise-linear model in the bootstrap distribution. C shows the lower 2.5% confidence boundary. Values are smaller where fewer samples are available.
(EPS)

Text S1 Text S1 describes how the distribution of samples available for different amplitude and angle difference combinations potentially influences fixation duration estimates.
(PDF)
