# Supplementary material for: Saccadic Momentum and Facilitation of Return Saccades Contribute to an Optimal Foraging Strategy
Source: PLoS Comput Biol. 2013 Jan 17;9(1):e1002871. doi: 10.1371/journal.pcbi.1002871 (PMC3547797; doi:10.1371/journal.pcbi.1002871)
Supplement: Text S1 — describes how the distribution of samples available for different amplitude and angle difference combinations potentially influences fixation duration estimates. (PDF) [file pcbi.1002871.s002.pdf]

Supplementary materials

At first glance one might suspect that some remaining structure is present in fixation durations that are corrected with the piecewise linear model (Figure 4E).

However, comparing Figure 4E and Figure 2B (top left) shows that the remaining pattern in 4E is correlated with the number of samples available per bin. We suspected that this and the typically non-normal distribution of fixation durations might introduce statistical effects that could make it likely to observe the remaining pattern.

To estimate the size of such a hypothetical effect, we created a baseline distribution of fixation durations that takes different sample sizes into account by jointly shuffling the angle and amplitude difference labels of corrected fixation durations. We then used this distribution as our null hypothesis that no angle and amplitude difference effect is present and compared the empirical fixation durations against it by bootstrapping (N=1750) confidence intervals for the null hypothesis. At an uncorrected 95% alpha level, we find that in the case of corrected fixation durations, 349 significant deviations (279 expected at p=0.05) occur. Correcting for multiple comparisons leaves only 14 significant deviations.

The significant deviations were spread out uniformly and did not form clusters. Furthermore, as expected, the expected bootstrapped duration per bin is close to zero for every bin. The size of the confidence intervals however varies systematically with the number of bins available per bin (see Figure S1).

Because of the above, it is plausible that the remaining pattern in the residuals is due to statistical effects introduced by unequal number of samples per bin and the non-normal distribution of fixation durations.
